# Pathogenic Relationships in Cystic Fibrosis and Renal Diseases: CFTR, SLC26A9 and Anoctamins

**DOI:** 10.3390/ijms241713278

**Published:** 2023-08-26

**Authors:** Karl Kunzelmann, Jiraporn Ousingsawat, Andre Kraus, Julien H. Park, Thorsten Marquardt, Rainer Schreiber, Björn Buchholz

**Affiliations:** 1 Physiological Institute, University of Regensburg, University Street 31, 93053 Regensburg, Germany; jiraporn.ousingsawat@vkl.uni-regensburg.de (J.O.); rainer.schreiber@ur.de (R.S.); 2 Department of Nephrology and Hypertension, Friedrich Alexander University Erlangen Nuremberg, 91054 Erlangen, Germany; andre.kraus@uk-erlangen.de (A.K.); bjoern.buchholz@uk-erlangen.de (B.B.); 3 Department of Pediatrics, University Hospital Münster, 48149 Münster, Germany; julien.park@ukmuenster.de (J.H.P.); thorsten.marquardt@ukmuenster.de (T.M.)

**Keywords:** TMEM16A, TMEM16F, anoctamin, SLC26A9, CFTR, pendrin

## Abstract

The Cl^−^-transporting proteins CFTR, SLC26A9, and anoctamin (ANO1; ANO6) appear to have more in common than initially suspected, as they all participate in the pathogenic process and clinical outcomes of airway and renal diseases. In the present review, we will therefore concentrate on recent findings concerning electrolyte transport in the airways and kidneys, and the role of CFTR, SLC26A9, and the anoctamins ANO1 and ANO6. Special emphasis will be placed on cystic fibrosis and asthma, as well as renal alkalosis and polycystic kidney disease. In essence, we will summarize recent evidence indicating that CFTR is the only relevant secretory Cl^−^ channel in airways under basal (nonstimulated) conditions and after stimulation by secretagogues. Information is provided on the expressions of ANO1 and ANO6, which are important for the correct expression and function of CFTR. In addition, there is evidence that the Cl^−^ transporter SLC26A9 expressed in the airways may have a reabsorptive rather than a Cl^−^-secretory function. In the renal collecting ducts, bicarbonate secretion occurs through a synergistic action of CFTR and the Cl^−^/HCO_3_^−^ transporter SLC26A4 (pendrin), which is probably supported by ANO1. Finally, in autosomal dominant polycystic kidney disease (ADPKD), the secretory function of CFTR in renal cyst formation may have been overestimated, whereas ANO1 and ANO6 have now been shown to be crucial in ADPKD and therefore represent new pharmacological targets for the treatment of polycystic kidney disease.

## 1. Introduction

Cystic fibrosis (CF) is a genetic disorder caused by variants in the cystic fibrosis transmembrane conductance regulator (CFTR) gene that affects approximately 90,000 people worldwide. The absence or impaired function of the CFTR protein is associated with dysfunction in several organs, particularly the respiratory and gastrointestinal tracts. Autosomal dominant polycystic kidney disease (ADPKD) is the most commonly inherited nephropathy. It is characterized by the development and enlargement of renal cysts due to increased cell proliferation, extracellular matrix abnormalities, and increased CFTR-mediated transepithelial fluid secretion. CFTR is also required for the excretion of bicarbonate in the renal collecting ducts. A defect in renal bicarbonate excretion can lead to systemic alkalosis.

A large number of reports describe various functional changes induced either by the knockout or knockdown or the overexpression of CFTR. Although CFTR is the essential Cl^−^ channel for transepithelial Cl^−^ secretion, many of the morphological and functional changes that occur when CFTR expression is altered are thought to be caused indirectly by the up- or downregulation of intracellular signaling pathways or by metabolic changes that affect the function of other, independent proteins. In this context, physical protein–protein interactions leading to functional coupling with partner proteins (e.g., the epithelial Na^+^ channel ENaC or the Cl^−^/HCO_3_^−^ exchanger pendrin) are reported to control ion transport and other cell and tissue functions. Large “interactomes” for CFTR now exist, leading to the somewhat provocative question of whether CFTR “interacts with everything” [1,2,3,4,5,6]. Indeed, CFTR is a true hub for kinases and the crosstalk of cAMP and Ca^2+^ [7,8]. In this brief review, we focus specifically on the interplay of CFTR with the Cl^−^ transporter SLC26A9, the Ca^2+^-activated Cl^−^ channel anoctamin 1 (ANO1), and the phospholipid scramblase anoctamin 6 (ANO6) in the airways and kidneys.

## 2. CFTR Causes Constitutive Basal Cl^−^ Secretion in the Airways

The airways show basal Cl^−^ secretion in the absence of secretagogues, i.e., cAMP- or Ca^2+^-dependent stimulation, raising the question which of the above Cl^−^ channels and transporters are actually responsible for basal secretion. It should be noted that no spontaneous basal CFTR activity was observed in patch clamp recordings in the absence of PKA- or PKC-dependent stimulation. Previous studies provided conflicting data on the origin of basal Cl^−^ secretion. While some studies suggested spontaneous activity of CFTR as the cause of basal Cl^−^ secretion [9,10,11,12], other studies proposed SLC26A9 as the responsible transporter [13,14,15,16,17]. Until now, separating the two pathways (CFTR and SLC26A9) has been difficult because (i) there have been no specific inhibitors for SLC26A9, (ii) CFTR inhibitors do not target specifically [18], and (iii) intracellular transport and activity of SLC26A9 depend on the expression and function of CFTR [16,19,20,21,22].

In the study by Jo et al., the SLC26A9 inhibitor S9-A13 had no inhibitory effect on airway Cl^−^ transport either in vitro or ex vivo, whereas CFTRinh172 inhibited both basal and cAMP-induced Cl secretion [23]. Interestingly, neither inhibition of adenosine receptors nor inhibition of adenylate cyclase blocked basal Cl^−^ secretion, raising questions about additional mechanisms for the activation of CFTR. Thus, it is possible that increased protein kinase C (PKC) activity, e.g., through ATP release and binding to purinergic receptors, keeps part of the CFTR active at basal cAMP levels and PKA phosphorylation [24,25,26,27]. The Hanrahan lab has also recently demonstrated expression of SLC26A4 (Pendrin) in primary nasal and bronchial ciliated epithelial cells, which enhances Cl^−^ secretion through the stimulation of CFTR [28]. The molecular mechanism of STAS/R domain interaction has been previously shown for the activation of CFTR by SLC26A6 [29]. As discussed in the next section, the Ca^2+^-activated Cl^−^ channel ANO1 may contribute to the maintenance of basal CFTR activity through a Ca^2+^-dependent mechanism.

## 3. Relationship between CFTR and Anoctamins

Early studies showed cAMP/PKA and increases in intracellular Ca^2+^ as two independent second messenger pathways that lead to epithelial Cl^−^ secretion [30]. The pharmacological tools used to discriminate between both Cl^−^ conductances, however, are rather non-specific, and thus our team could not clearly keep these conductances apart [31,32]. We and others also reported that CFTR seemingly “inhibits” endogenous Ca^2+^-activated Cl^−^ currents (CaCC) in *Xenopus* oocytes, bovine pulmonary artery endothelium cells and isolated parotid acinar cells [33,34,35,36,37]. After molecular identification of the Ca^2+^-activated Cl^−^ channel (CACC) as anoctamin 1 (ANO1), it was found that CFTR does not inhibit ANO1 and that ANO1 currents and CFTR currents are not additive, i.e., they do not add up to the sum of both currents [38,39].

In fact, Ca^2+^-enhancing agonists such as purinergic or muscarinic ligands mostly activate CFTR-dependent secretion in the airways [24,40,41], while ANO1 currents rapidly inactivate it due to the mechanisms outlined in previous reports [42,43,44,45]. Therefore, after inhibition or in the absence of CFTR, CACC is very short-lived, and in the intestine, there is not even an apical CACC (ANO1) [45,46,47,48]. The rapid inactivation of ANO1 is reasonably well understood [42,49] and raises the question as to whether direct pharmacological activation of ANO1 by the synthetical compound ETX001 (called ETD-002 in the clinical trial) can be successful in restoring Cl secretion in the airways of CF patients [50,51]. It should also be noted that ANO1 is expressed at only very low levels in the airways [52].

The activation of ANO1 in CF may even be counterproductive, as ANO1 is a proinflammatory factor which enhances mucus production and mucus secretion (at least in our hands) and supports pain sensation [53,54,55,56,57]. Moreover, during inflammatory airway diseases such as asthma and CF, ANO1 is upregulated in pulmonary arterial vessels, where it supports airway constriction [52,58,59,60,61,62,63,64,65]. Finally, ANO1 supports the release of inflammatory cytokines such as IL-8 and the accumulation of pulmonary CD-45 positive cells [53]. A phase 1 clinical trial with the ANO1-activator ETD-002 finished more than a year ago, but so far, no outcome has been reported. Because of the partially conflicting studies, further studies are required in animals with clear pulmonary inflammation and increased expression of ANO1. While pure cell culture studies are insufficient, the F508del-piglet model might be very helpful in determining the true role of ANO1 in human airways, particularly under inflammatory conditions.

## 4. Crosstalk between CFTR and ANO1

The previous section provokes the question whether there exists a crosstalk between CFTR and ANO1. Studies reported attenuated expression of ANO1 in the apical membrane of airway epithelial cells, when coexpressed with F508del-CFTR [31,66]. We found evidence for an interaction between ANO1 and CFTR through PSD-95/Dlg/ZO-1 (PDZ) domain proteins, as described for SLC26A9 [16]. The functional interaction between ANO1 and CFTR is based on the crosstalk of intracellular Ca^2+^ and the intracellular cAMP signaling pathway. Crosstalk is facilitated by exchange proteins directly activated by cAMP (EPAC1) and Ca^2+^-sensitive adenylate cyclase type 1 (ADCY1). The assembly of such a local signalosome also depends on the presence of G-protein coupled receptors (GPCRs) [32,67,68]. In the next chapter, we will show that a functional interaction of CFTR and ANO1 also exists at the level of the membrane expression of CFTR.

## 5. Reduced Plasma Membrane Expression of CFTR in the Absence of ANO1

**Figure 1:**
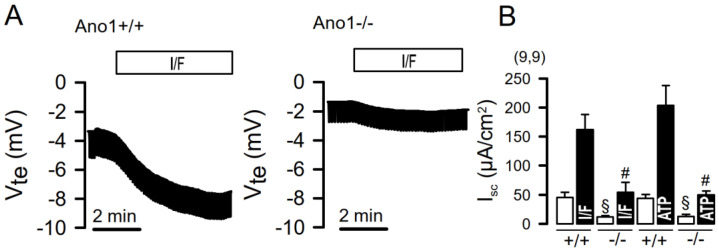
Attenuated CFTR-dependent Cl^−^ secretion in mice with intestinal epithelial knockout of Ano1. (**A**) Original Ussing chamber recordings obtained from colonic epithelia under open circuit conditions, as described in [31]. Stimulation of colonic epithelia with IBMX and forskolin (I/F; 100 µM/2 µM) induced a pronounced voltage deflection in normal mouse colonic epithelium (Ano1+/+), indicating pronounced cAMP-activated secretion. The voltage deflection, i.e., Cl^−^ secretion, was strongly attenuated in a colonic tissue obtained from a mouse lacking expression of Ano1 (Ano1−/−). (**B**) Calculated equivalent short circuit currents (Isc) indicate strongly attenuated CFTR-dependent (I/F-stimulated) as well as Ca^2+^-dependent (100 µM ATP-stimulated) Cl^−^ secretion in mouse colon lacking epithelial expression of Ano1 (−/−), when compared to wild-type colons (−/−). Mean ± SEM (number of experiments for +/+ and −/−). ^§^ Significantly reduced when compared to basal Isc in +/+ tissues (ANOVA). ^#^ Significantly reduced when compared to stimulated Isc in +/+ tissues (ANOVA). For methods, see [31].

Cell-specific knockout of ANO1 in ciliated airway epithelial cells abolished Ca^2+^-activated Cl^−^ currents and largely reduced Ca^2+^-dependent Cl^−^ secretion in mouse airways. Moreover, Ca^2+^-dependent Cl^−^ transport was abolished in intestinal epithelial cells from epithelial-specific ANO1-knockout mice [31]. However, we reported the surprising observation that in parallel to the loss of ANO1-dependent transport, CFTR-dependent Cl^−^ transport was also lost in these ANO1-knockout animals [31] (Figure 1). In both the airways and the intestine, we found that the expression of CFTR in the apical membrane was largely attenuated, if not abolished. It should be noted that the expression of ANO1 in mouse airways is very low, while clear expression of ANO1 is detected in colonic epithelial cells, mainly located in the basolateral membrane [69,70].

How are these findings explained? From earlier studies, we know that ANO1 tethers the endoplasmic reticulum (ER) near the plasma membrane (PM) via binding to the inositol trisphosphate receptor (IP_3_R). Due to this, IP_3_-mediated Ca^2+^-release from the ER and store-operated Ca^2+^ influx are strongly improved in airway sub-apical or colonic sub-basolateral membrane compartments (please note that in the large intestine, ANO1 is located primarily near or in the basolateral membrane [71,72,73]). Along this line, it is of note that extended synaptotagmin-1 (ESYT1), another ER-PM tether, was found to further enhance PM expression of ANO1 and Ca^2+^ signaling [74]. PM expression of CFTR requires exocytosis, which is enhanced by the higher local sub-membranous Ca^2+^ levels facilitated by ANO1 [75]. Moreover, exocytosis also depends on ANO6 [75,76]. In knockout mice for ANO1 and ANO6 and in a number of human cell lines, we and others showed that both ANO1 and ANO6 are important for the PM insertion and activation of CFTR [52,53,75,76,77,78,79,80]. In this context, Ca^2+^-dependent activation of PKC could play a role [24,81,82]. Enhanced sub-membranous Ca^2+^ may further support CFTR activity via Ca^2+^- activated adenylate cyclases and EPAC. Finally, both anoctamins are equally important for mucus secretion by goblet cells and the release of lysozyme and other antimicrobial factors by Paneth cells. We speculate that ANO1 facilitates local Ca^2+^ signaling and not, or at least not primarily, Cl^−^ secretion. Evidence for this will be provided in the next chapter outlining data obtained from the first two patients that lacked expression of functional ANO1.

## 6. A Loss of Function Mutation of ANO1 in Patients Also Abolished CFTR-Mediated Cl^−^ Transport

**Figure 2:**
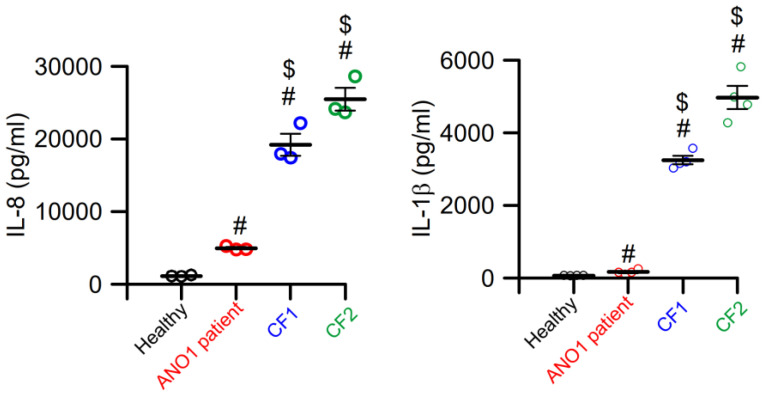
Cytokines in a sputum sample from a patient carrying the ANO1 loss-of-function variant c.897 + 3_897 + 6delAAGT are strongly reduced when compared to samples from CF patients. Sputum samples were obtained from a healthy volunteer, the patient carrying the ANO1 variant c.897 + 3_897 + 6delAAGT, and from two CF patients, and concentrations of the cytokines IL-8 and IL-1ß were determined. Although the ANO1 patient lacks CFTR function in addition to the defect in ANO1 Ca^2+^-dependent Cl^−^ secretion, cytokines were strongly reduced when compared to CF patients carrying known CFTR mutations. Mean ± SEM (number of measurements). ^#^ Significantly enhanced when compared to the healthy volunteer (ANOVA). ^$^ Significantly enhanced when compared to the ANO1 patients (ANOVA). For methods see [84].

The first two patients expressing the ANO1-variant c.897 + 3_897 + 6delAAGT were reported recently. These patients expressed a dysfunctional ANO1 and lack of Ca^2+^-activated Cl^−^ currents [83]. The two reported siblings presented in early infancy with reduced intestinal peristalsis and recurrent episodes of hemorrhagic diarrhea. Analysis of isolated primary airway epithelial cells obtained from one of the patients reproduced the results obtained earlier in tissue-specific ANO1-knockout mice [31]. Apart from the absence of Ca^2+^-activated Cl^−^ transport, CFTR Cl^−^ currents were also completely absent, possibly due to a lack of expression of CFTR in the apical membrane. Moreover, analysis of cells obtained from a heterozygous sibling showed reduced Cl^−^ secretion [83]. Rather surprisingly, the patients did not show a CF-like lung phenotype, although sweat tests were positive, indicating defective CFTR Cl^−^ conductance. This is even more surprising given the fact that both Ca^2+^-activated ANO1 and cAMP-activated CFTR Cl^−^ conductances were absent. Cytokine levels measured in sputum samples obtained from one of the ANO1 patients were largely reduced when compared to the cytokine levels measured in samples from two CF patients (Figure 2). While this may provide further evidence for the pro-inflammatory role of ANO1 [57], it also raises questions regarding the true contribution of apical Cl^−^ conductance for CF pathology [54].

## 7. Contribution of CFTR and ANO1/ANO6 to Regulated Cell Death

In previous chapters, we reported the functional relationship between CFTR and ANO1. Here, we will elaborate on the relationship between CFTR and ANO6, a Ca^2+^-activated phospholipid scramblase that is also permeable to ions [85]. Prior to the era of CFTR, the so-called intermediate conductance outwardly rectifying Cl^−^ channel ICOR (ORCC, ORDIC) was shown in many reports to be the essential epithelial secretory Cl^−^ channel that is absent in cystic fibrosis [86,87,88,89,90,91,92], while others identified the ICOR as a patch clamp artifact occurring during membrane excision [93]. After its identification in 1989 [94,95,96], CFTR was considered to be the real secretory Cl^−^ channel [81], and later it became clear that there was a regulatory relationship between CFTR and ICOR/ORCC [97,98]. ORCC has since been described as an apoptosis-related Cl^−^ channel, but its molecular identity still remains unclear [99]. After the identification of the anoctamin family, we reported CFTR as being an activator of ANO6, and we demonstrated that ANO6 is a core component of ICOR involved in apoptotic cell death [85,100]. Thus, the phospholipid scramblase ANO6 found a role in CFTR-dependent cell death and a place within the pathogenic relationships of CFTR with other proteins [101].

CFTR has been proposed to release glutathione (GSH) from airway epithelial cells to be enriched in the apical airway surface liquid, which will neutralize reactive oxygen species (ROS) [102,103,104]. Apparently, GSH efflux does not change cytosolic GSH content [105], and we were therefore unable to detect different ROS levels depending on the expression of CFTR [106]. However, we observed an enhanced activity of ANO6 in the presence of wtCFTR. As in most other cell types, ANO6 is also expressed in airway epithelial cells, where it can scramble plasma membrane phospholipids, which leads to cell death [85]. The importance of ANO6 for regulated cell death is also demonstrated in ANO6 knockout mice. In these animals, the number of apoptotic cells within the intestinal epithelium was drastically reduced [106].

In vivo inoculation with *P. aeruginosa* or *Staphylococcus aureus* induced lipid peroxidation in the lungs of CFTR-knockout mice and in wild-type animals. Exposure of human airway epithelial cells to *P. aeruginosa* induced an increase in reactive oxygen species (ROS) and caused lipid peroxidation and cell death. *P. aeruginosa*-induced cell death was independent of expression of wt-CFTR or F508del-CFTR [107]. In contrast, knockout of ANO1 clearly reduced cell death, possibly because ANO1 supports Ca^2+^-dependent activation of ANO6 and thus phospholipid scrambling [108]. Notwithstanding these results, good evidence exists for enhanced oxidative stress in the lungs of people with CF, while ROS have been shown to directly activate ANO1 and ANO6, providing another functional link between a lack of CFTR function, anoctamins and cell death [44,109,110].

## 8. Airway Secretion of Bicarbonate (HCO_3_^−^) by CFTR

Disruption of CFTR in mice causes organ diseases typical of cystic fibrosis (CF), such as meconium ileus, distal intestinal obstructions with mucus accumulation, blockage of pancreatic ducts and lacrimal gland dilatation, along with some developmental defects [111]. These initial studies were confirmed in a number of subsequent transgenic models for cystic fibrosis [112,113]. However, a central aspect of CF pathology, namely the chronic inflammatory airway disease, was hardly detectable in CF mice, and pH values in the airway surface liquid were not different [113]. However, in a number of studies with a CF pig model, the human CF pathology could be nicely reproduced [114,115]. In contrast to transgenic F508del-cftr mice, CF pigs demonstrated reduced airway surface pH, impaired bacterial killing and adhesive mucus that disrupts mucociliary transport [116,117,118,119]. It was concluded that dysfunctional CFTR leads to a lack of HCO_3_^−^ secretion, thus causing acidification of the airway surface liquid (ASL), followed by mucus abnormalities, attenuation of airway defenses, inflammation and a typical CF lung phenotype [118,119,120]. However, in another porcine CFTR-knockout model, acidic ASL pH could not be detected [12,121]. In this study, micro pH-electrode measurements were used to assess ASL pH directly in the small airways of lung sections from acutely sacrificed newborn piglets [12]. Pathological changes in these CFTR−/− lungs were not detected. Along this line, another study reported mucus accumulation preceding pulmonary infection in children with CF [122]. Moreover, using a novel luminescent technology integrated with fiberoptic probes, an acidic airway surface liquid pH could not be detected in children with cystic fibrosis [123]. Taken together, these findings may provoke the question as to whether HCO_3_^−^ does also use another secretory pathway that is different to CFTR.

## 9. SLC26A9 Is Expressed in the Apical Membrane of Airways from CFTR-Knockout Piglets, but Not in Airways Expressing CFTR-F508del

SLC26A9 is one out of eleven proteins of the SLC26A family of anion transporters. It is expressed in the gastrointestinal tract, the respiratory system, male tissues and skin, and may have different functions depending on the organ in which it is expressed. Variants of SLC26A9 were associated with an increased incidence of meconium ileus and diabetes in patients with cystic fibrosis (CF) [124,125,126,127,128,129]. While there is good evidence for the role of SLC26A9 in gastrointestinal transport, it remains unclear whether it affects CF lung disease severity and airway responses to CFTR therapeutics [130,131]. Recently, the expression of the Cl^−^/HCO_3_^−^ exchanger SLC26A9 was found to be absent in the apical membrane of human F508del-CFTR/F508del-CFTR airways [132], which corresponds to the well-known inhibitory effect of F508del-CFTR on the membrane expression of SLC26A9 [16,21]. In contrast, SLC26A9 was found to be well expressed in the apical membrane of airway epithelial cells in non-CF lungs and in lungs from CFTR-knockout piglets [132]. Plasma membrane expression of SLC26A9 in the absence of CFTR was also shown in cell cultures, whereas coexpression with F508del-CFTR abrogates the biosynthesis, trafficking and function of SLC26A9 [14,16,21]. We therefore speculate that the normal ASL pH measured in airways of CFTR−/− piglets is due to the normal location and function of SLC26A9, which suggests that HCO_3_^−^ can be secreted by SLC26A9 to the luminal side of the airways.

**Figure 3:**
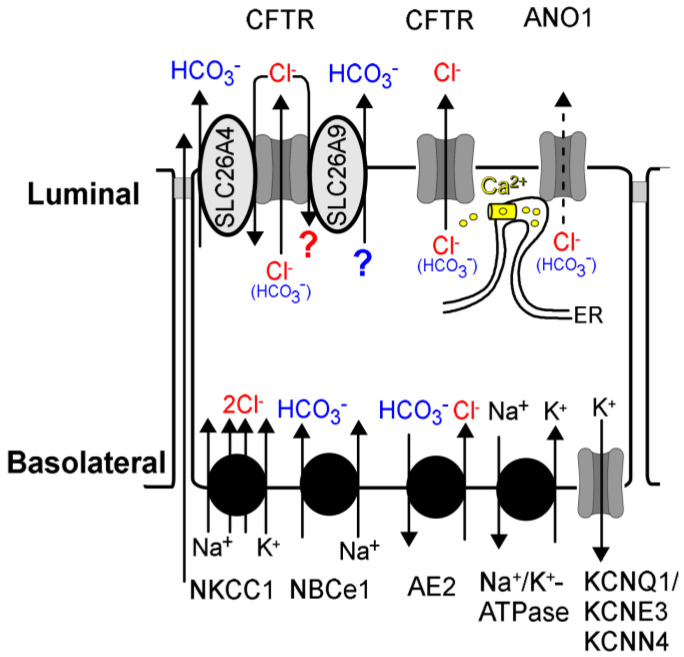
Proposed modified scheme for Cl^−^ and HCO_3_^−^ secretion in airways. Cl^−^ and HCO_3_^−^ ions are taken up on the basolateral site of airway epithelial cells by the Na^+^/K^+^/2Cl^−^ cotransporter NKCC1, NBCe1, and AE2, respectively. The Na^+^/K^+^-ATPase and the basolateral K^+^ channels KCNQ1/KCNE3 and KCNN4 provide the driving force for luminal anion secretion. Luminal secretion of Cl^−^ occurs essentially through CFTR, with only a negligible contribution of ANO1. ANO1 supports Cl^−^ secretion through tethering of the endoplasmic reticulum near the apical compartment and an increase in the subapical Ca^2+^ concentration that facilitates activation and membrane expression of CFTR. CFTR also provides a recycling pathway for Cl^−^ that allows the secretion of HCO_3_^−^ by SLC26A4 and probably SLC26A9.

The transport of HCO_3_^−^ by SLC26A9 has been proposed in some studies [133,134,135], but was not found by other laboratories [13,136,137,138]. Additional species-specific tissue factors like epithelial polarization or coexpression with additional proteins like CFTR may affect SLC26A9 transport function. Along this line, the vast majority of SLC26A9 expressed in non-polarized cells remains in the cytosol, while it is nicely expressed in the apical membrane of polarized cells [23]. Coexpression with wtCFTR or the complete absence of CFTR (CFTR knockout piglets) allows the proper plasma membrane location of SLC26A9, in contrast to the airways of CF-patients expressing a F508del-CFTR allele [132]. Like other SLC26A proteins (SLC26A3,4,6,8), SLC26A9 may also interact physically with CFTR via R (regulatory) and STAS (Sulphate Transporter and AntiSigma factor antagonist) domains, and probably through PDZ-domain interaction [13,29,139,140,141,142,143]. A recent study showed a contribution of SLC26A9 to airway bicarbonate secretion using the novel SLC26A9 inhibitor S9-A13. Online recordings of ASL pH in primary human nasal epithelial cells under thin film conditions indicated a sustained decrease in ASL pH caused by S9-A13, while subsequent activation of CFTR was unable to re-alkalinize ASL pH [23]. These initial results require confirmation by additional studies in vivo to clearly define the role of SLC26A9-dependent bicarbonate transport in airways (Figure 3). It will also be interesting to learn to what extent SLC26A9 contributes to airway HCO_3_^−^ secretion when compared to SLC26A4 (pendrin), which probably secretes the most HCO_3_^−^, particularly during inflammation [28]. In conclusion, the main task of SLC26A9 in the airways and particularly in the alveoli could actually be the reabsorption of Cl^−^ rather than Cl^−^ secretion (reviewed in [144]).

## 10. Bicarbonate Is Secreted in Renal Collecting Ducts, Which Requires CFTR, Pendrin and Possibly ANO1

**Figure 4:**
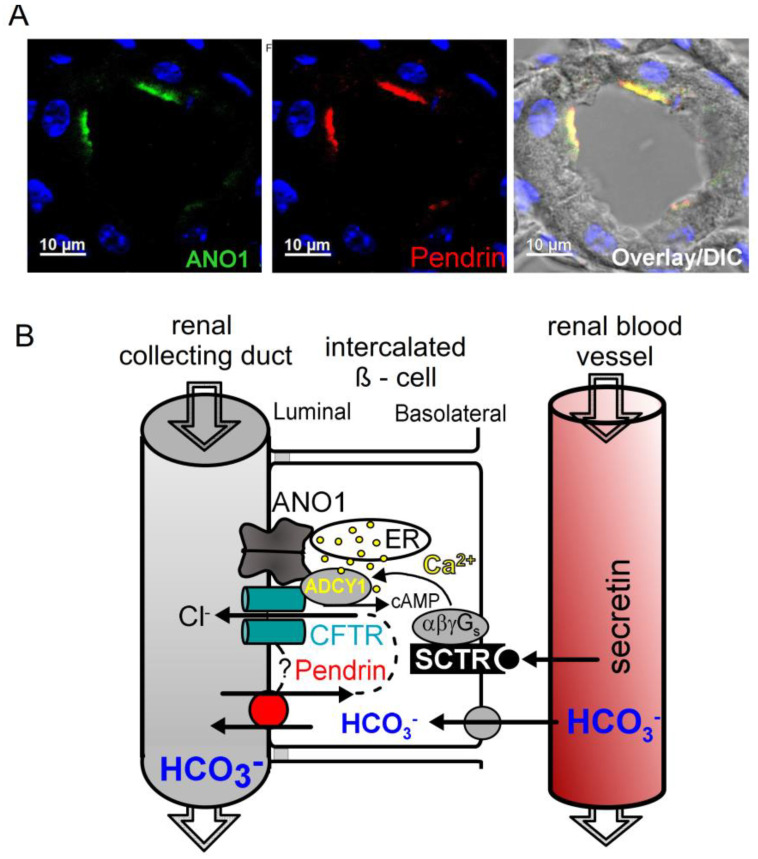
ANO1 is colocalized with pendrin in the apical membrane of ß-intercalated cells. (**A**) Immunocytochemistry demonstrating colocalization of ANO1 and pendrin in the apical membrane of ß-intercalated cells of mouse collecting ducts. For methods, see [150]. (**B**) Model showing the molecular mechanisms for HCO_3_^−^ excretion by collecting duct ß-intercalated cells. Blood HCO_3_^−^ is taken up into ß-intercalated cells and is transported by pendrin into the collecting duct lumen in exchange with Cl^−^, which is recycled via colocalized CFTR. In addition, CFTR may directly interact with SLC26A4. Colocalized ANO1 tethers the endoplasmic reticulum (ER) to the apical membrane and facilitates efficient Ca^2+^ signaling in the apical compartment, which supports insertion of CFTR into the apical membrane and its activation. An increase in blood secretin leads to the activation of basolateral secretin receptors (SCTR), which further activates CFTR and HCO_3_^−^ excretion. For methods, see [150].

CFTR is also expressed in the tubular epithelial cells of the human kidneys, where it affects different transport functions. Early studies suggested a role of CFTR for renal bicarbonate (HCO_3_^−^) transport [145,146], which was later confirmed for many other epithelial organs [147,148,149]. HCO_3_^−^ excretion was found to be largely reduced in people with CF, particularly when patients were challenged with the hormone secretin, which binds to its receptor and increases intracellular cAMP. A defect in renal bicarbonate excretion can lead to metabolic alkalosis occasionally observed in CF patients. Detailed studies in mice lacking expression of CFTR or the HCO_3_^−^ transporter SLC26A4 (pendrin) finally uncovered the molecular mechanism [150,151]. Physical interaction of CFTR with pendrin and/or Cl^−^ recycling via CFTR drives the tubular release of HCO_3_^−^ through apical pendrin and urinary excretion. This process takes place in ß-intercalated cells of the renal collecting duct, which coexpress CFTR, pendrin and receptors for secretin [150]. ANO1 is colocalized together with pendrin (and CFTR) in the apical membrane of renal ß-intercalated cells and may support the activity of CFTR [150] (Figure 4).

## 11. CFTR and ANO1 in Polycystic Kidney Disease: Which One Counts?

While CF and CF-associated metabolic alkalosis are rare, autosomal dominant polycystic kidney disease (ADPKD) is the most common monogenic kidney disease, affecting approximately 1 in 1000 individuals, often resulting in end-stage renal disease [152]. Mutations in either the PKD1 (~78%) or PKD2 (~15%) gene [153] cause the formation of multiple renal cysts which originate from renal tubule epithelial cells, predominantly the principal cells of the collecting duct [154,155]. The cysts grow continuously over years and cause compression of the adjacent intact nephrons, resulting in a decline of renal function [156]. Two key features are identified for cyst growth: a change from an absorptive to a secretory epithelium and the abnormal proliferation of cyst epithelial cells [157]. It is assumed that the major secretory force for cyst fluid secretion is apical cAMP-dependent Cl^−^ secretion, and several studies have suggested CFTR as the essential Cl^−^ channel [158,159,160].

However, recently, Cabrita et al. demonstrated that cyst growth in ADPKD is prevented by pharmacological and genetic inhibition of the calcium-activated chloride channel ANO1 [161]. Loss of PKD1 increased the expression of ANO1 and CFTR and induced Cl^−^ secretion in murine kidneys. Importantly, upregulated ANO1 enhanced intracellular Ca^2+^ signaling and the proliferation of PKD1-deficient renal epithelial cells. In contrast, increases in Ca^2+^ signaling, cell proliferation and CFTR expression were not observed in PKD1/ANO1 double knockout mice. In a sophisticated renal collecting duct M1 cell organoid model and in primary renal epithelial cells, cell proliferation and Cl^−^ secretion were also dependent on enhanced expression of ANO1 [162,163]. Knockdown of PKD1 or PKD2 increased basal intracellular Ca^2+^ levels and enhanced purinergic Ca^2+^ release from the endoplasmic reticulum. Ca^2+^ signals, proliferation, and Cl^−^ secretion were largely reduced via the knockdown or blockade of ANO1. ANO1 is therefore central to enhanced Ca^2+^ release from IP_3_-sensitive ER Ca^2+^ stores, and is a central player in ADPKD caused by mutations in PKD1 and PKD2. The data strongly suggest that pharmacological inhibition of ANO1 slows down the progression of ADPKD.

Concerning disease progression, male gender is a major risk factor [164,165]. Talbi et al. found that kidneys from PKD1 knockout mice had a more pronounced phenotype in males compared to females. The proliferation of cells from the cyst epithelium was enhanced in male when compared to female kidneys. This was paralleled by higher basal intracellular Ca^2+^ concentrations in cells isolated from PKD1 knockout males. These results again suggest enhanced intracellular Ca^2+^ levels contributing to enhanced proliferation and cyst development in male kidneys. Notably, the incubation of renal cells with dihydrotestosterone enhanced basal Ca^2+^ levels and ATP-stimulated ANO1 currents [166]. Similar results were obtained in a mouse model for autosomal recessive polycystic kidney disease (ARPKD) [157,167]. Finally, polycystic kidneys are under constant oxidative stress, which causes lipid peroxidation and the activation of ANO1 and ANO6 [44,110,168]. Therefore, inhibition of anoctamins may be a new avenue for therapeutic intervention in ADPKD.

## 12. Targeting ANO1 or CFTR in ADPKD?

Inhibitors of Cl^−^ currents such as diphenylamine-2-carboxylate and knockdown of CFTR by antisense oligo-nucleotides inhibited cAMP-activated Cl^−^ currents in cyst cells [169]; 8807590). CFTRinh-172 or Ph-GlyH-101 reduced the cyst growth of renal MDCK cells in a metanephric mouse kidney model and a rapidly progressive neonatal Pkd1 knockout mouse model [159,160]. In three CF patients with concomitant ADPKD, disease progression was delayed when compared to their siblings without CF [170,171]. However, the ADPKD-protective effect provided by CF was not confirmed in a subsequent report [172], and CFTR expression in isolated ADPKD cyst cells was shown to be very heterogeneous [158,169,173]. It is therefore not entirely clear whether inhibiting CFTR slows down cyst progression. Moreover, both CFTRinh-172 or Ph-GlyH-101 have pronounced off-target effects and affect intracellular Ca^2+^ signals which actually inhibit ANO1 [18].

Initial studies showed that Ca^2+^-activated ANO1 Cl^−^ currents contribute to cyst growth [174]. ATP is released by cyst cells, accumulates in the cyst lumen and activates ANO1 via the stimulation of purinergic receptors [155,174,175]. By contrast, the scavenging of ATP by apyrase, the P2Y2 receptor antagonist suramin, and the knockdown of P2Y2 inhibited cyst growth [155]. These studies and the subsequent work outlined above [161,162] suggested ANO1 as the relevant pharmacological target to inhibit in ADPKD.

**Figure 5:**
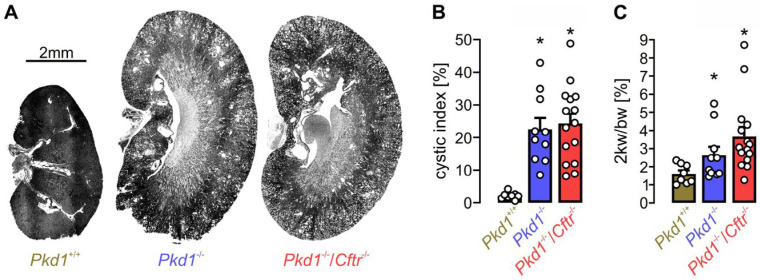
Knockout of Cftr does not affect cyst growth in an ADPKD mouse model. KspCreER^T2^; Pkd1^lox;lox^ mice (Pkd1^−/−^; *n* = 10) and KspCreER^T2^; Pkd1^lox;lox^/Cftr^lox;lox^ mice (Pkd1^−/−^/Cftr^−/−^; *n* = 15) received daily intraperitoneal injections of tamoxifen (2 mg/kg body weight dissolved in 5% ethanol and 95% neutral oil at postnatal days 20–22) to induce tubule-specific deletion of Pkd1 or co-deletion of Pkd1 and Cftr. Non-induced KspCreER^T2^; Pkd1^lox;lox^ mice (Pkd1^+/+^; *n* = 8) served as controls. Analyses were performed 10 weeks after induction with tamoxifen. (**A**) Representative kidney sections at the end of the experiment. For methods, see [161]. (**B**) Analysis of the cystic indices defined as the ratio of the cortical cystic area divided by the whole cortex area. (**C**) Two-kidney weight per body weight ratio. No significant effect of CFTR-knockout was found [161]. Bars show means ± SEM, and dots indicate individual values. * Significant increase compared to Pkd1^+/+^ (*p* < 0.05; one-way ANOVA). For methods, see [176].

Taken together, in mouse studies, ANO1 is a dominant driver of secretion-dependent cyst enlargement, while we also found that knockout of CFTR had no significant impact on cyst growth [176] (Figure 5). Nevertheless, it is important to keep in mind that the physiological contribution of ANO1 in mice is probably greater while the contribution of CFTR is lower than in humans. In mice, CFTR shows lower activity in the airways but has a more pronounced contribution to intestinal transport [177]. In the kidneys of healthy mice, CFTR is only clearly expressed in ß-intercalated cells, where it controls HCO_3_^−^ secretion [150]. Interestingly, a recent report showed that the application of the CFTR-corrector VX-809 (Lumacaftor) in a Pkd1 knockout and the Pkd1^RC/RC^ mouse model reduced cyst growth [178,179]. These findings were explained by a cellular translocalization of CFTR and the Na^+^/H^+^ exchanger 3. A clinical phase 2 placebo-controlled randomized trial investigated the efficacy and safety of the CFTR corrector GLPG2737 in ADPKD patients (NCT04578548) [180]. More studies are required to analyse the contribution of ANO1 to cyst formation in human tissue. The central aspects of ANO1 include its obvious pro-proliferative and de-differentiating properties [101], which after all may have a larger impact on cyst progression than fluid secretion.

## 13. Inhibitors of ANO1

Given the promising results obtained through the genetic and pharmacological inhibition of ANO1, ANO1 qualifies as a potential target for the treatment of ADPKD. ANO1 function can be addressed by drugs that have already been approved for other indications, like niclosamide or benzbromarone [161]. Niclosamide is an essential oral anthelminthic drug used for decades to treat parasitic infections, but it is meant for short-term use [181]. Benzbromarone is a uricosuric drug that has been used in the treatment of gout over the last 30 years. Although withdrawn by Sanofi for safety reasons after reports of hepatotoxicity, it is still marketed in several countries by other drug companies (drugs.com/international/benzbromarone.html (accessed on 27 June 2023)). Hepatotoxicity is rare and occurs in 1 in 17,000 patients [182]. For comparison, the only drug approved for treatment of ADPKD, the vasopressin-2-receptor antagonist tolvaptan, has a hepatotoxic risk of 1 in 3000. Therefore, many experts in the field have questioned the withdrawal of benzbromarone [182].

## 14. Conclusions

This review summarizes recent findings on CFTR, SLC26A9, and anoctamin 1 and 6 in the airways and kidneys. It becomes clear that these ion channels and transports do not stand alone, but rather operate in a functional and metabolic network and should therefore be analyzed in the context of their molecular and functional interactions. However, data obtained in different animal and cell culture models often cause confusion due to the differential expression of proteins in the different species, tissues or cell lines. This is particularly evident for the differences in CFTR expression in human, piglets and mice, and even more so for ANO1, which is almost absent in native airways but abundant in cultured airway cells. The authors believe that progress in understanding cystic fibrosis and polycystic kidney disease can only be achieved by considering data from animal studies. 

The current trend towards over-regulated and lengthy animal welfare applications should be corrected. Pure cell culture studies, even when performed on primary cells in differentiated culture, carry the risk of misinterpretation. This is particularly important in the development of new pharmacological strategies. Because of the increasingly complex pathogenic relationships, new therapeutic strategies must first be thoroughly evaluated in animal studies before they can be applied in humans.

## Authors Contributions

Conceptualization, K.K., J.O., A.K., R.S. and B.B.; methodology., J.O., A.K., J.H.P., T.M., R.S. and B.B.; formal analysis, J.O., A.K. and B.B.; writing—original draft preparation, K.K., R.S. and B.B.; writing—review and editing, J.O., A.K., J.H.P., T.M., R.S. and B.B. All authors have read and agreed to the published version of the manuscript.

## Data Availability

Not applicable.
